# No relationship between left ventricular radial wall motion and longitudinal velocity and the extent and severity of noncompaction cardiomyopathy

**DOI:** 10.1186/1476-7120-10-9

**Published:** 2012-03-19

**Authors:** Kadir Caliskan, Osama I Soliman, Attila Nemes, Ron T van Domburg, Maarten L Simoons, Marcel L Geleijnse

**Affiliations:** 1 Thoraxcenter, Erasmus MC, Rotterdam, the Netherlands; 2 Throaxcenter, Room Bd577, Erasmus MC,'s-Gravendijkwal 230, 3015, CE Rotterdam, the Netherlands

**Keywords:** Noncompaction cardiomyopathy, LV function, Heart failure, Tissue doppler imaging

## Abstract

**Background:**

Noncompaction cardiomyopathy (NCCM) is characterized by a prominent trabecular meshwork and deep intertrabecular recesses. Although systolic dysfunction is common, limited information is available on differences in wall motion of the normal compacted and noncompacted segments. The purpose of this study was to assess radial wall motion and longitudinal wall velocity in patients with NCCM, according to the extent and severity of noncompaction.

**Methods:**

The study comprised 29 patients in sinus rhythm (age 41 ± 15 years, 15 men), who fulfilled stringent diagnostic criteria for NCCM and compared to 29 age and gender matched healthy controls. Segmental radial wall motion of all compacted and noncompacted segments was assessed with the standard visual wall motion score index and longitudinal systolic (Sm) wall velocity with tissue Doppler imaging of the mitral annulus. For each LV wall a normalized Sm value was calculated. The extent and severity of NC in each LV segment was assessed both in a qualitative and quantitative manner.

**Results:**

Heart failure was the primary clinical presentation in half of the patients. NCCM patients had a wall motion score index of 1.68 ± 0.43 and a normalized Sm of 82 ± 20%. The total and maximal noncompaction scores were not related to the wall motion score index and the normalized Sm. NCCM patients with and without heart failure had similar total and maximal noncompaction scores.

**Conclusions:**

In NCCM patient's radial wall motion and longitudinal LV wall velocity is impaired but not related to the extent or severity of noncompaction.

## Background

Noncompaction of the left ventricle (LV) or noncompaction cardiomyopathy (NCCM), is a relatively new clinico-pathologic entity, first described by Engberding and Bender in 1984 [1]. It is characterized by a prominent trabecular meshwork and deep intertrabecular recesses communicating with the LV cavity and is thought to be caused by an arrest of normal embryogenesis of the myocardium [2,3]. The noncompacted (NC) LV segments often show abnormal wall motion. However, NCCM may be a part of a more generalized cardiomyopathy, involving both the morphologically normal and abnormal LV segments. Unfortunately, still limited information is available on differences in wall motion of the normal compacted (C) and abnormal NC segments [4-6]. Therefore, the purpose of this study was to assess radial wall motion and longitudinal wall velocity in patients with NCCM, according to the extent and severity of NC.

## Methods

### Study population

The study comprised 29 consecutive patients in sinus rhythm (age 41 ± 15 years, 15 men), who fulfilled the following stringent diagnostic criteria for NCCM, as described by Jenni et al [7].

**Figure 1:**
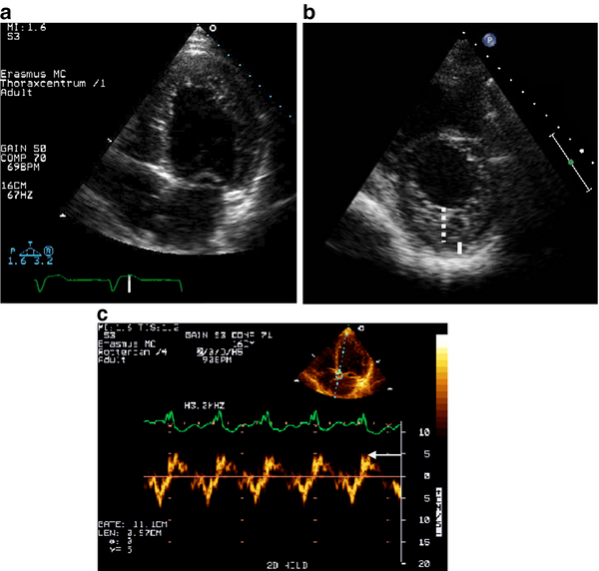
**Echocardiographic features of a 58-years-old male with chronic heart failure due to familial noncompaction cardiomyopathy; (**a**) the apical 4-chamber view shows extensive trabeculations, especially in the apical and lateral LV walls**. (**b**) The parasternal short axis view in end-systole, the NC/C ratio is > 2 (respectively dashed line and small bar). (**c**) Low septal Sm (normal value 8.3 ± 1.5 cm/s)

1. An excessively thickened LV myocardial wall with a two-layered structure comprising a C layer on the epicardial side and a NC layer of prominent trabeculations and deep intertrabecular recesses on the endocardial side (Figure 1).

2. A NC/C myocardial thickness ratio > 2 measured at the moment of maximal thickness in end-systole at the parasternal short axis (Figure 1).

3. Color-Doppler evidence of deep intertrabecular recesses in communication with the LV cavity.

4. Absence of coexisting cardiac anomalies (eg hypertension, coronary artery disease, valvular or congenital heart disease).

### Radial LV wall motion

According to the recommendations of the American Heart Association on standardized myocardial segmentation and nomenclature for tomographic imaging of the heart, a 17-segment model was used [8] Radial wall motion of all C and NC LV segments was assessed using the standard wall motion score (1 = normal motion, 2 = hypokinetic, 3 = akinetic, 4 = dyskinetic). Global LV function was subsequently expressed in terms of a wall motion score index. No measurements of LV volumes and ejection fraction were made because of the inherent problem to identify the endocardial border in the presence hypertrabeculation.

### Longitudinal LV wall motion

Tissue Doppler imaging was applied by placing the sample volume at the side of the mitral annulus in apical 4, 2, and 3-chamber views. Gain and filter settings were adjusted as needed to eliminate background noises and to allow for a clear tissue signal. To acquire the highest tissue velocities the angle between the Doppler beam and the longitudinal motion of the investigated structure was adjusted to a minimal level. The systolic velocities of the mitral annulus (Sm) were recorded end-expiratory at a sweep speed of 75 or 100 mm/s and measured using electronic calipers with EnConcert software (Philips, Best, and The Netherlands). For each patient, the average of three measurements was calculated. Normal Sm values for the posteroseptal (8.3 ± 1.5 cm/s), anterolateral (9.4 ± 0.6 cm/s), anterior (8.8 ± 1.6 cm/s), inferior (9.1 ± 1.8 cm/s), inferolateral (9.6 ± 0.6 cm/s), and anteroseptal (7.3 ± 1.3 cm/s) LV walls were derived from 29 for age and gender matched healthy controls (mean age 43 ± 7 year, 15 men) without hypertension or diabetes, and with normal left atrial and LV function and morphology. Subsequently, for each LV wall a normalized Sm value was calculated as: wall specific Sm in NCCM patient/wall specific Sm in control subjects × 100%.

### Extent and severity of noncompaction

The extent and severity of NC in each LV segment was assessed quantitatively by measuring the NC and C myocardial wall thickness with electronic calipers. A severity score was calculated for each LV segment by one experienced observer (KC): 2 points were given if noncompaction was clear with prominent trabeculations present (NC/C ratio ≥ 2), 1 point was given if prominent trabeculations were present but not fulfilling the Jenni criteria (NC/C ratio > 1.0 but < 2.0). In addition, from these quantitative measurements the most prominent noncompacted segment with the highest (i.e. maximal) NC/C ratio was identified in each of the 6 individual LV walls (excluding the apical cap).

The data are collected and analyzed in accordance with hospital institutional review board policies.

### Statistical analysis

Descriptive data for continuous variables are presented as mean ± SD. Continuous data were compared with the Student *t *test. Linear regression analysis with Pearson's correlation was performed to examine the relationship between the radial and longitudinal LV wall motion and the extent and the severity of noncompaction. A 2-sided P value < 0.05 was considered statistically significant. For all analysis, commercially available software package was used (Prism 5, GraphPad Software Inc., http://www.graphpad.com).

## Results

The clinical and echocardiographic data of the 29 patients with typical features of NCCM are summarized in Table 1. Heart failure was the primary clinical presentation in half of the patients. In the majority of the cases (*n *= 18 (62%), the NCCM was familial.

**Table 1:** Clinical and echocardiographic characteristics of all patients

Age, years	41 ± 15
Male, n (%)	15 (52)
Presentation, n (%)	
Heart Failure	16 (55)
Arrhythmias	5 (17)
Screening	5 (17)
Other	3 (10)
NYHA, n (%)	
I	13 (45)
II	11 (38)
III	5 (17)
IV	0
Left atrium, mm	38 ± 7
LV end-diastolic diameter, mm	53 ± 7
LV end-systolic diameter, mm	40 ± 8
Interventricular septum, mm	9 ± 2
Fractional shortening, %	25 ± 9
Wall motion score index	1.68 ± 0.43
PA systolic, mm Hg	25 ± 6
Noncompacted segments, %	50 ± 15
Absolute mean Sm ± SD, cm/s	7.1 ± 1.6
Normalized mean Sm, %	82 ± 20

Values in mean ± standard deviation or numbers (%)

### Radial wall motion

**Figure 2:**
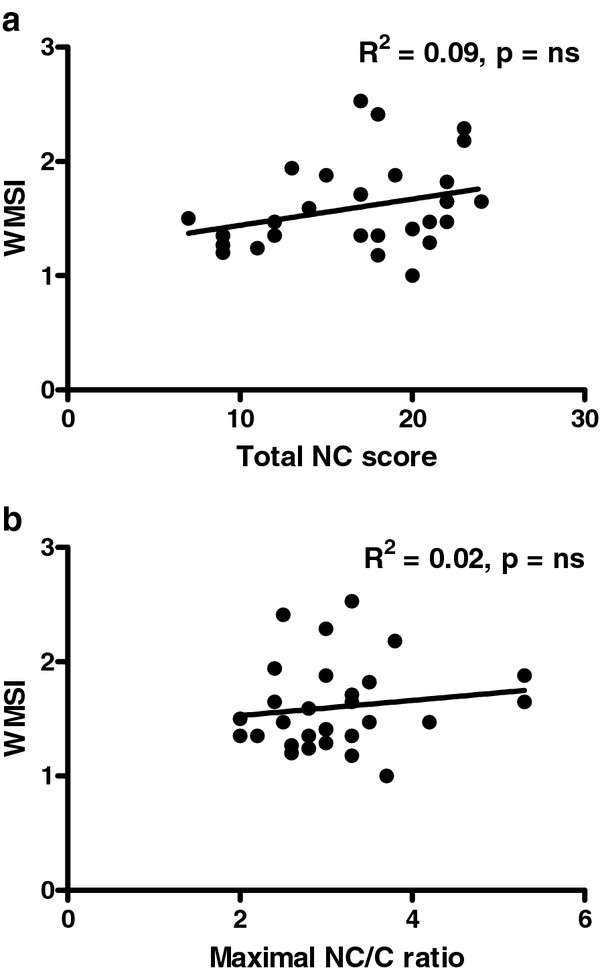
**Relation between the wall motion score index and the total noncompaction score (2a), and the maximal noncompaction score (2b)**.

Interobserver agreement for segmental analysis of radial wall motion between two observers (KC and MLG) was 76% in both noncompacted and compacted LV segments with a kappa values of 0.60 and 0.56, respectively. NCCM patients had a wall motion score index of 1.68 ± 0.43. The total and maximal NC/C ratio scores were not related to the wall motion score index (R^2 ^0.09 and 0.02 respectively) (Figures 2a and 2c).

### Longitudinal wall motion

**Figure 3:**
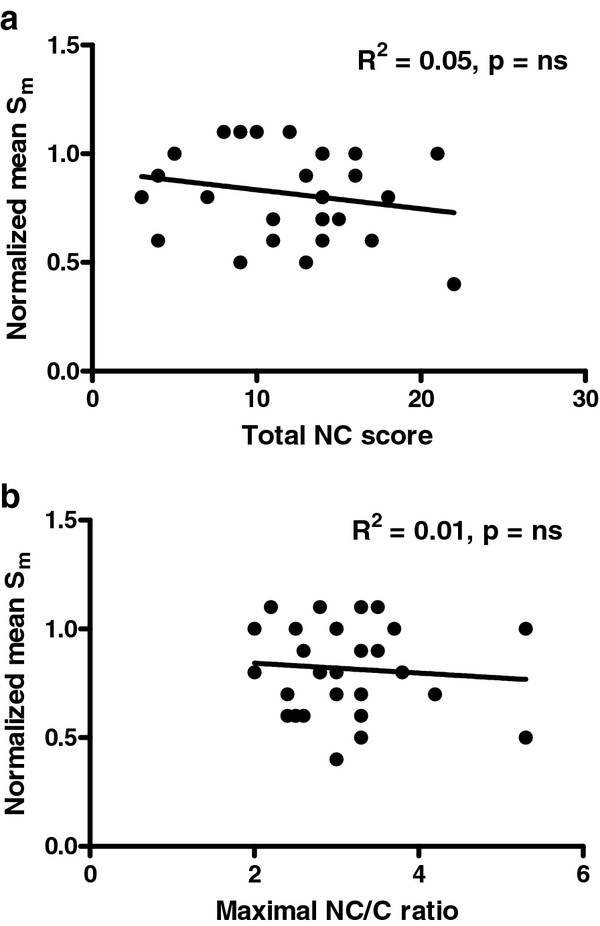
**Relation between the mean normalized systolic mitral annular velocities (Sm) and the total noncompaction score (3a), and the maximal noncompaction score (3b)**.

**Figure 4:**
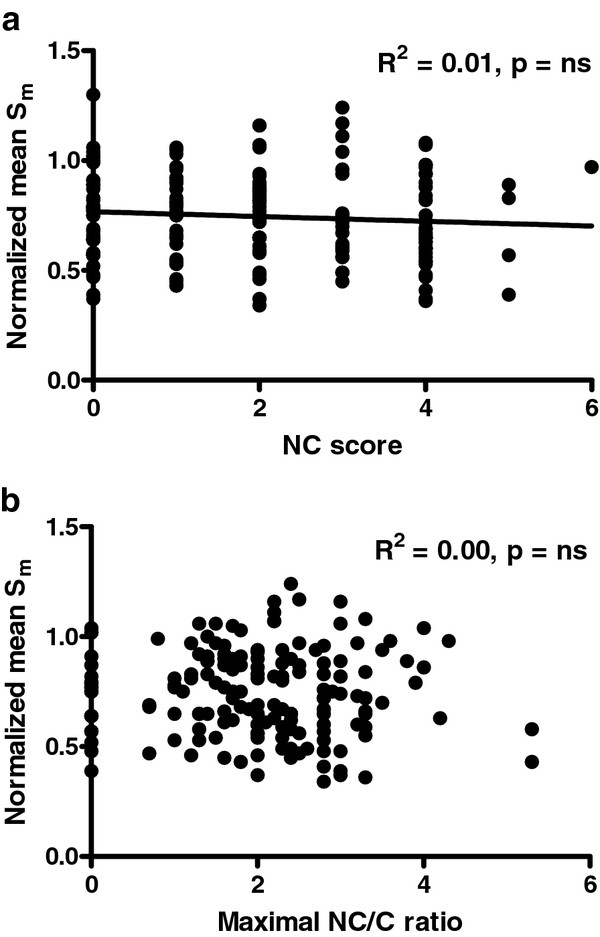
**Relation between normalized systolic mitral annular velocities (Sm) and the total noncompaction score (4a), and the maximal noncompaction score (4b) in individual LV walls**.

NCCM patients had a normalized Sm of 82 ± 20%. The total and maximal NC scores were not related to the normalized Sm (R^2 ^0.02, 0.05 and 0.01 respectively) (Figures 3a and 3b and 4a and 4b).

### NCCM patients with versus patients without heart failure

All parameters of systolic LV function (fractional shortening, wall motion score index, normalized Sm) were significantly lower in NCCM patients with heart failure (Table 2). However, no differences were seen between NCCM patients with and without heart failure in the total and maximal NC scores.

**Table 2:** Clinical and echocardiographic data of patients with and without heart failure

	Heart failure N = 16	No heart failure N = 13	*P*-value
Age, years	44 ± 14	37 ± 15	ns

Male, n (%)	9 (56)	6 (46)	ns

LBBB, n (%)	3	0	ns

LVH, n (%)	2	3	ns

Left atrium, mm	38 ± 6	38 ± 7	ns

LV end-diastolic diameter, mm	55 ± 7	51 ± 8	ns

LV end-systolic diameter, mm	43 ± 8	36 ± 7	0.02

Interventricular septum, mm	10 ± 2	8 ± 2	ns

Fractional shortening, %	22 ± 8	30 ± 7	0.01

Wall motion score index,	1.75 ± 0.40	1.39 ± 0.21	0.01

PA systolic, mm Hg	28 ± 7	22 ± 4	0.02

Noncompacted segments, %	49 ± 15	52 ± 15	ns

Normalized mean Sm, %	75 ± 20	93 ± 16	0.02

Values in mean ± standard deviation or numbers (%)

LBBB: left bundle branch block, LVH: left ventricular hypertrophy on ECG

## Discussion

The main finding of this study is that in patients with NCCM both radial and longitudinal LV wall motion is impaired but not related to the extent and severity of noncompaction. The extent and severity of noncompaction was also not related to systolic dysfunction or HF symptom presentation, in line with the previous publications and confirming that the cardiomyopathy in NCCM is not regional but global problem [4,6].

According to the last AHA scientific statement NCCM is classified as a primary, genetic cardiomyopathy [9]. The distinct phenotype of cardiomyopathy fits probably within the spectrum of abnormalities triggered by sarcomere gene defects [10-12]. The most common presentation in NCCM patients is systolic heart failure, less frequent presentations include ventricular arrhythmias and thrombo-embolic complications, including cerebro-vascular accidents en peripheral emboli [3,13-15].

The NC segments in NCCM patients often show abnormal wall motion [4]. However, NCCM may be a part of a more generalized cardiomyopathy, involving both the morphologically normal and abnormal segments. As described before in other studies [4] the wall motion score index was abnormal in NCCM patients, and both the NC and C segments showed abnormal wall motion. However, our study is the first to demonstrate that there is no relation between the extent and severity of NC and wall motion. It should be noted that visually studying wall motion is problematic because of its subjective nature. However, interobserver segmental agreement was near-identical in noncompacted and compacted LV segments (76% versus 76% with kappa values of 0.60 and 0.56, respectively). [16] In our opinion, measurement of LV volumes and ejection fraction is not an alternative because of the inherent problems of the technique and the impossibility of tracing the true endocardium because of the trabecular structures. To better elucidate global and regional LV function we measured longitudinal LV function with tissue Doppler imaging. The advantage of assessment of mitral annular velocities is that the region of interest from which the measurements are taken (the mitral annulus) is not involved in the process of NC but the measurements reflect function of walls involved in the process of NC. Regional longitudinal LV function was impaired, confirming previous findings by us on regional volume changes assessed by three-dimensional echocardiography, although in the patients without heart failure it was quite normal. Importantly, regional longitudinal LV function was impaired irrespective of the extent and severity of NC. Interestingly, our study confirms recent findings by Tufekcioglu et al. that NCCM patients with heart failure show more abnormal parameters of systolic LV function but not a greater involvement of NC [5]. These data further support our findings. This implies also that for example the extent and severity of NCCM could not been used for prediction LV dysfunction and/or heart failure in individual patients and that the patho-physiology of the LV dysfunction/heart failure in NCCM yet to be defined.

More definite answers on dysfunction of C versus NC LV segments should come from speckle tracking echocardiographic strain and strain rate studies [17], although due to the nature of the NC myocardium (with a very difficult fibre orientation) calculation of regional deformation may be difficult [18,19].

Previously, the role of tissue Doppler imaging has been shown in establishing the diagnosis of HCM in patients with LVH and permitting the early identification of subclinical myocardial abnormalities of contraction and relaxation velocities, before hypertrophy is manifest [20]. This may be also relevant to the asymptomatic NCCM patients and relatives and yet to be studied.

The main limitations of this study is the small numbers of the study populations, the methods used to assess the left regional left ventricular function and absence of long -term follow up data correlating the tissue Doppler imaging and clinical outcomes.

## Conclusions

In NCCM patient's radial wall motion and longitudinal LV wall velocity is impaired but not related to the extent or severity of noncompaction cardiomyopoathy. Both affected (noncompacted) and seemingly non-affected (compacted) segments contribute to reduced LV function in this cardiomyopathy. This suggests that the LV dysfunction in NCCM is not regional but global problem.

## Competing interests

The authors declare that they have no competing interests.

## Supplementary Material

Additional file 1 **Movie 1 A Apical four chamber and parasternal short axis view of a 37 years old male presenting with severe heart failure en left bundle branch block**. Familial screening revealed several affected first degree relatives. Echocardiographically, there are prominent trabeculations with noncomapcted/compacted ratio > 2 at the parasternal short axis view in end systole. There is not only visual LV dyssynchrony, but also diffuse wall motion abnormalities which are not only confined to the noncompacted segments. The systolic wall velocity with tissue Doppler imaging of the mitral annulus was respectively 4.8 cm/s (age/gender matched healthy control: 9.13 cm/s) and 6.3 cm/s (control: 9.4 cm/s) in septal wall and lateral wall. Click here for file

Additional file 2 **Movie 1 B Apical four chamber and parasternal short axis view of a 37 years old male presenting with severe heart failure en left bundle branch block**. Familial screening revealed several affected first degree relatives. Echocardiographically, there are prominent trabeculations with noncomapcted/compacted ratio > 2 at the parasternal short axis view in end systole. There is not only visual LV dyssynchrony, but also diffuse wall motion abnormalities which are not only confined to the noncompacted segments. The systolic wall velocity with tissue Doppler imaging of the mitral annulus was respectively 4.8 cm/s (age/gender matched healthy control: 9.13 cm/s) and 6.3 cm/s (control: 9.4 cm/s) in septal wall and lateral wall. Click here for file
